# Serum microRNA Profiles Serve as Novel Biomarkers for Autoimmune Diseases

**DOI:** 10.3389/fimmu.2018.02381

**Published:** 2018-10-16

**Authors:** Fangfang Jin, Huanhuan Hu, Ming Xu, Shoubin Zhan, Yanbo Wang, Huayong Zhang, Xi Chen

**Affiliations:** ^1^State Key Laboratory of Pharmaceutical Biotechnology, Jiangsu Engineering Research Center for MicroRNA Biology and Biotechnology, School of Life Sciences, NJU Advanced Institute for Life Sciences, Nanjing University, Nanjing, China; ^2^Department of Rheumatology and Immunology, The Affiliated Drum Tower Hospital of Nanjing University Medical School, Nanjing, China

**Keywords:** autoimmune disease, Treg, serum, miRNA, biomarker

## Abstract

Autoimmune diseases involve a complex dysregulation of immunity. Autoimmune diseases include many members [e.g., rheumatoid arthritis (RA) and systemic lupus erythematosus (SLE)], and most of them are classified according to what organs and tissues are targeted by the damaging immune response. Many studies have focused on finding specific biomarkers for single autoimmune diseases, but so far, there are no universal biomarkers for detecting almost all autoimmune diseases. Serum miRNAs have served as potential biomarkers for detecting various diseases. The purpose of this study was to find a universal biomarker for diagnosing autoimmune diseases. Regulatory T cells (Tregs) play a crucial role in protecting an individual from autoimmunity, and depletion of Tregs in mice is considered a representative animal model of autoimmune disease. Two mouse models for Treg depletion, in which Treg was depleted by CD25mAb (in C57 mice) or by diphtheria toxin (DT) (in Foxp3^DTR^ mice), were investigated, and 381 miRNAs were identified in the serum of mice with Treg depletion. A distinctive circulating miRNA profile was identified in Treg-depleted mice and in patients with autoimmune disease. QRT-PCR confirmation and ROC curve analysis determined that six miRNAs (miR-551b, miR-448, miR-9, miR-124, miR-148, and miR-34c) in the Treg-depleted mouse models and three miRNAs [miR-551b (specificity 73.5%, sensitivity 88.4%), miR-448 (specificity 82.4%, sensitivity 91.3%), and miR-124 (specificity 76.5%, sensitivity 91.3%)] in patients with RA, SLE, Sjogren's syndrome (SS), and ulcerative colitis (UC) could serve as valuable specific biomarkers. These circulating miRNAs may represent potential universal biomarkers for autoimmune diseases diagnosis and prognosis.

## Introduction

Autoimmune diseases reflect the interplay between environment and genetic factors (1–3). The diseases share a substantial degree of immunopathology, including increased secretion of inflammatory cytokines by autoreactive CD4^+^ T cells and a loss of Regulatory T cells (Tregs) function (4, 5). Most autoimmune diseases are classified based on which organs and tissues are targeted by the damaging immune response [e.g., primary biliary cirrhosis (6), type 1 diabetes mellitus (1), arthritis (7), and myositis (8)]. Autoimmune diseases include many types, and there is an autoimmune disease specific to nearly every organ in the body (8). Clinically, specific diagnostic methods are used for each autoimmune disease, which is tedious and costly. Therefore, it is urgent to find a universal marker to diagnose autoimmune diseases, which will provide new possibilities for autoimmune disease detection and treatment.

Tregs, characterized by expressing CD4, CD25, and Forkhead box P3 (Foxp3) transcription factor, play pivotal roles in protecting an individual from autoimmunity. These roles have been identified in mice with Treg depletion or absence, which results in the development of autoimmune gastritis, thyroiditis, multiple sclerosis (MS), type 1 diabetes, ankylosing spondylitis (AS), inflammatory bowel disease (IBD), systemic lupus erythematosus (SLE), rheumatoid arthritis (RA), Sjogren's syndrome (SS), and ulcerative colitis (UC) (4, 9–11). Thus, Tregs depletion in mice is considered a representative animal model of autoimmune disease. Two approaches are typically used to deplete Tregs in mice. In the first, Treg cells are depleted by constitutive expressing CD25. Previous studies have demonstrated that injection of depleting antibodies directed against CD25 will lead to a mild autoimmune disease (11–13). The other method is to use Foxp3^DTR^ mice, which are created by designing a construct in which cDNA encoding the diphtheria toxin receptor (DTR) is inserted into the 3′ untranslated region (3′UTR) of Foxp3. After continuous injection of DT, Treg cells are efficiently depleted, which affects multiple organs and leads to fatal autoimmune pathology (14, 15).

microRNAs (miRNAs) are small regulatory RNA molecules that function to regulate gene expression and play vital roles in various physiologic and pathologic processes. Our study (16) and others' studies (17–21) have found that serum miRNAs can serve as potential biomarkers for detecting a variety of diseases, including immune diseases. Song et al. (22) found that circulating miRNAs play a key role in diagnosing congenital heart defects (CHD) and predicting CHD risk in offspring. Sharaf-Eldin et al. (23) determined that three miRNAs (miR-326, miR-223, and miR-145) expression profiles are promising diagnostic biomarkers for SLE and MS. Anaparti et al. (24) indicated miR-103a-3p as a prognostic biomarker for preclinical RA. Guo et al. (25) found that miRNA expression patterns are different in inflamed and noninflamed terminal ileal mucosa of patients with Crohn's disease (CD), and dysregulated miRNAs may be responsible for CD pathogenesis. According to current knowledge, immunosuppression relies partly on Tregs and involves in autoimmune disease and cancer, but the serum miRNA profiles of these diseases are less similar (26–28). Therefore, we deem that an investigation of serum miRNA profiles in immunodeficient animal models and patients with autoimmune diseases can be an easy and insightful pathway to provide valuable diagnostic and therapeutic approaches in the future.

In this study, we established two animal models of Treg depletion by using CD25 mAb in C57 mice and DT in Foxp3^DTR^ mice. miRNA low density array and quantitative reverse-transcription PCR (qRT-PCR) confirmation were used to characterize the miRNA expression profiles in serum of Treg-depleted mice. ROC curve analysis determined that six miRNAs (miR-551b, miR-448, miR-9, miR-124, miR-148, and miR-34c) could serve as valuable biomarkers for distinguishing Treg-depleted mice from controls. Then, we identified them in the serum from healthy controls, RA, SLE, SS, and UC patients. We found that three miRNAs (miR-448, miR-124, and miR-551b) could serve as novel diagnostic indicators and thereby provide some useful information about the molecular pathogenesis of autoimmune diseases.

## Materials and methods

### Animals

All animal experiments were performed in accordance with the National Institutes of Health Guide for the Care and Use of Laboratory Animals. Male 6–8-weeks-old C57BL/6J mice were purchased from the Model Animal Research Center of Nanjing University (Nanjing, China). The Foxp3^DTR^ mice were generously provided by Prof Alexander Rudensky (Memorial Sloan-Kettering Cancer Center, New York). The mice were maintained under specific pathogen-free conditions at Nanjing University.

### Reagents

TRIzol LS Reagent was purchased from Invitrogen. The mouse Treg staining kit#1 was purchased from eBioscience. DT from corynebacterium diphtheria was purchased from Sigma-Aldrich. The purified rat anti-mouse CD25 antibody was purchased from BD Pharmingen. A peripheral blood lymphocyte isolation kit was purchased from Tianjin Haoyang Biological Company.

### Depletion of tregs

In C57BL/6J mice, Tregs were transiently depleted by intraperitoneally injecting 0.5 mg purified rat anti-mouse CD25 antibody as we previously described (29). For Foxp3^DTR^ mice, frozen DT stocks were thawed once and 50 μg/kg of DT was injected intraperitoneally unless otherwise noted. To maximum the efficiency of CD4^+^ CD25^+^ Foxp3^+^ Treg elimination, we conducted injections every day for 7 consecutive days.

### Flow cytometric analysis

Peripheral blood and spleen were collected and analyzed by FACScalibur for CD4, CD25, and Foxp3 T cell expression as previously described (29). The results were analyzed by BD FACScalibur device.

### Measurement of cytokine levels in serum

Whole blood of mice was collected without anticoagulant and centrifugated to obtain serum. The levels of TNF-α, IL-6, and IFN-γ in serum were detected with ELISA kits (R&D) following the instructions as we previously described (29, 30).

### miRNA microarray

A minimum of 0.1 μg of total RNA was added to the GenoExplorer microRNA Expression System (GenoSensor Corporation, Tempe, AZ) containing probes in triplicate for mature miRNAs. miRNA concentrations are presented as threshold cycle (Ct). Significant differentially expressed miRNAs between the groups were analyzed and normalized to internal controls PC-U6B, U6-337, 5S-rRNA, and PC-HU5S recommended by the manufacturer. The relative concentration was calculated by the comparative Ct method (2^−ΔΔCt^). miRNAs were considered upregulated/downregulated if their Ct-values were <35 in the control samples and their levels in the Treg-depleted samples showed at least a 2-fold increase/decrease compared to the controls.

### Patients and healthy controls

The serum samples were collected according to protocols approved by the Medical Ethics Committee of Nanjing Drum Tower Hospital. All the RA patients are in the active stage of disease and received disease-modifying antirheumatic drugs (DMARDs), such as methotrexate (MTX), Leflunomide (LEF), Hydroxychloroquine (HCQ). Among them, three patients received glucocorticoids (GC) 5–15 mg/days. The Disease Activity Score with 28 joint (DAS28) of RA is 5.42 ± 1.83. In SLE patients, the Systemic Lupus Erythematosus Disease Activity Index (SLEDAI) is 14.28 ± 5.8, all of them have received hormone immunosuppression and the average dosage was 25 mg/days. Healthy controls are in normal physiological conditions and show no sign of pathologic factors after health examination. The ages of healthy individuals are matched with patients. The demographic characteristics of patients and healthy controls are listed in Supplemental Tables 1–8.

### Serum RNA isolation and qRT-PCR

Total RNA of serum was extracted using TRIzol LS Reagent (Invitrogen) following the instructions. qRT-PCR was performed on a LightCycler 480 real time PCR System (Roche, Mannheim, Germany) using TaqMan miRNA probes (Applied Biosystems) according to the instructions as we previously described (16, 31, 32).

### Statistical analysis

All data are representative of at least three independent experiments. All assays were performed in triplicate, and each experiment was repeated several times. Statistical analysis was performed using the *t*-test, when the groups >2, one way ANOVA followed by Bonferroni's multiple comparisons test were used. Data are presented as the means ± SEMs of at least three independent experiments. Differences were considered statistically significant at *P* < 0.05.

## Results

### Treg depletion by CD25 mAb or DT

**Figure 1:**
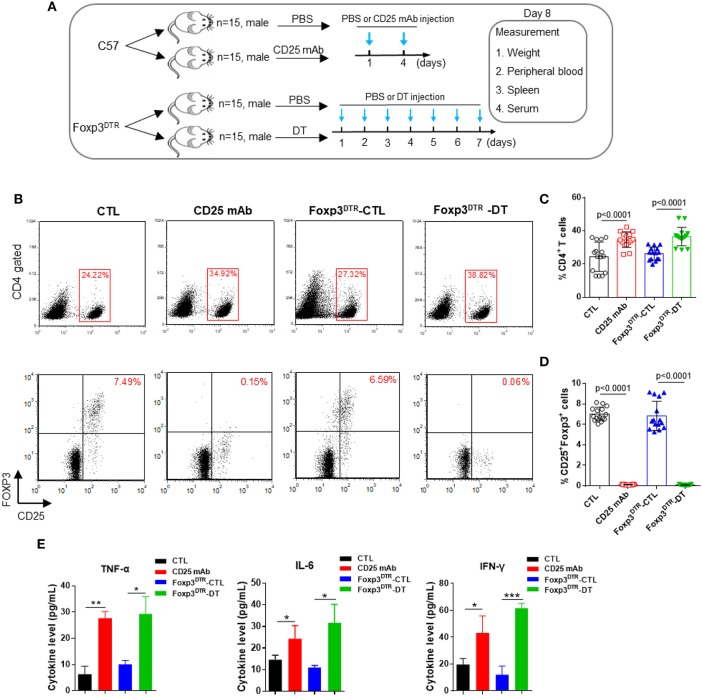
Anti-CD25 mAb and diphtheria toxin (DT) depletes CD4^+^ CD25^+^ Foxp3^+^ Treg cells. **(A)** Schematic diagram illustrating the experimental design. The C57BL/6 and Foxp3^DTR^ mice were divided into two groups (15 mice/group). Then, C57BL/6 mice were administered PBS or CD25 mAb every 3 days, while Foxp3^DTR^ mice were continuously injected with DT for 7 days. On day 8, all mice were sacrificed, peripheral blood, spleen, and serum were collected. **(B)** Analysis of CD4^+^ T cells and CD4^+^ CD25^+^ Foxp3^+^ Tregs in peripheral blood. **(C,D)** Statistical analysis of the percentages of CD4^+^ T cells and CD4^+^ CD25^+^ Foxp3^+^ Tregs in the mice of four groups. **(E)** Circulating TNF-α, IL-6, and IFN-γ levels in four groups of mice (*n* = 15). All the values are shown as the mean ± SEM. ^*^P < 0.05, ^**^P < 0.01, and ^***^P < 0.005.

First, C57BL/6 mice and Foxp3^DTR^ mice were injected with CD25 mAb or DT to eliminate CD4^+^ CD25^+^ Foxp3^+^ Tregs. To test the efficiency of Treg depletion, Treg levels were measured in peripheral blood and spleen on day 8 (Figure 1A). As shown in Figures 1B–D, CD4^+^ T cell levels increased after the CD25 mAb and DT injections. In peripheral blood, CD4^+^ CD25^+^ Foxp3^+^ Treg cells decreased significantly from 7.49 to 0.15% after CD25 mAb injection, and DT injection decreased the Treg levels from 6.59 to 0.06%. The levels of Treg were also reduced in spleen (Supplemental Figure 1A). In addition, mice depleted of Tregs weighed less than control mice (Supplemental Figures 1B,C). The levels of inflammatory cytokines TNF-α, IL-6, and IFN-γ were dramatically increased in serum from mice with Treg depletion compared to the mice without Treg depletion (Figure 1E), indicating that elimination of CD4^+^ CD25^+^ Foxp3^+^ Tregs is sufficient to disrupt immunological balance. These results suggest that Treg depletion can be used as a representative model of autoimmune diseases.

### Microarray analysis of serum miRNAs in treg-depleted mice and qRT-PCR confirmation of changed miRNAs

**Figure 2:**
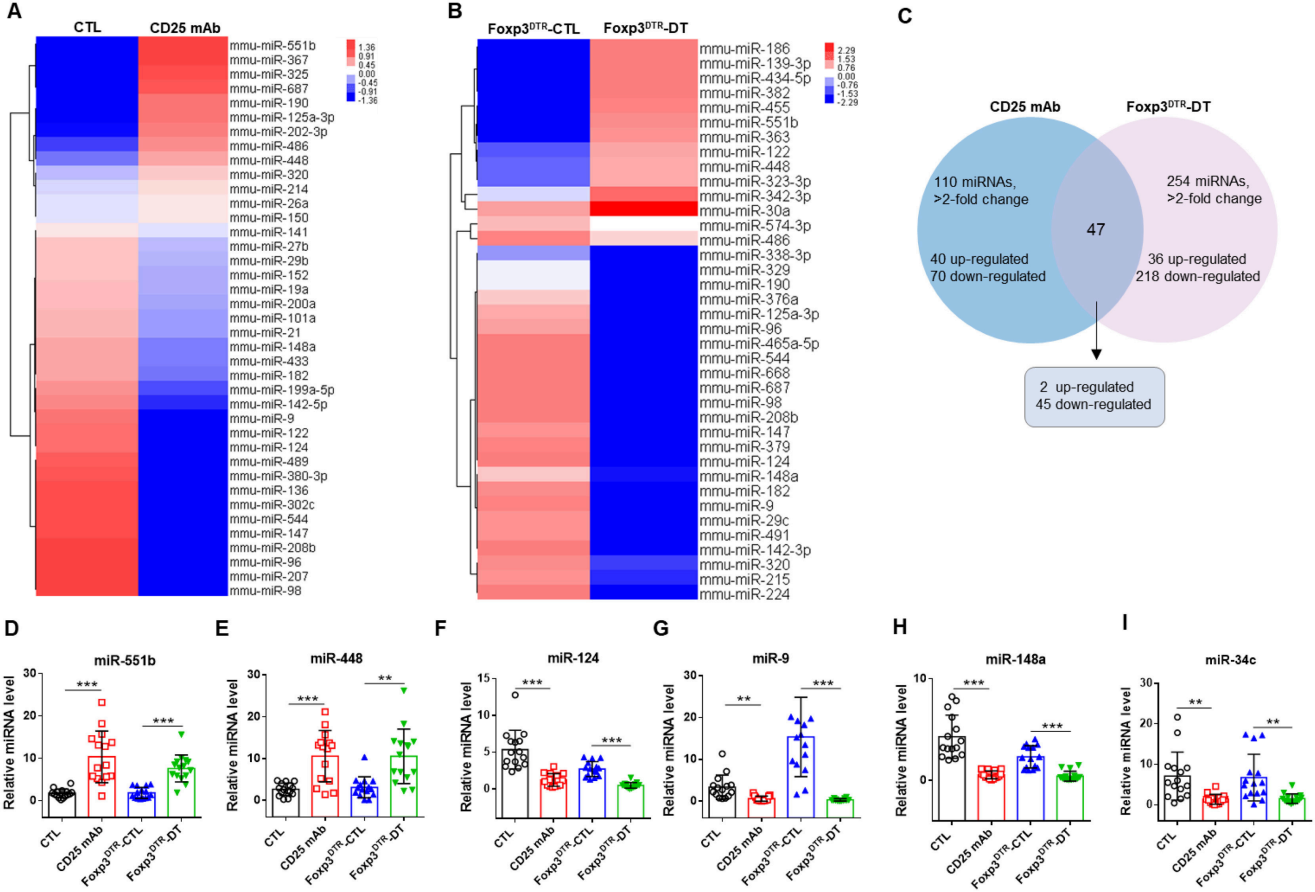
Hierarchical clustering of serum miRNA expression levels in Treg-depleted mice models. Hierarchical clustering of miRNAs differentially expressed in serum of mice from four groups: **(A)** CTL and CD25 mAb groups and **(B)** Foxp3^DTR^-CTL and Foxp3^DTR^-DT groups. **(C)** The changed miRNAs in Treg-depleted mice. **(D–I)** The relative levels of 6 selected serum miRNAs were studied in the mice from four groups. Serum samples from 15 mice in each group were pooled and subjected to qRT-PCR quantification. ^**^P < 0.01, and ^***^P < 0.005.

To identify the markedly changed serum miRNAs, we first analyzed the miRNAs differentially expressed between Treg-depleted and control mice by a TaqMan low density array. Of the 381 miRNAs scanned, 110 demonstrated >2-fold changes in the CD25 mAb group, 40 were upregulated and 70 were downregulated (Figures 2A,C). In the DT group, 254 miRNAs demonstrated >2-fold changes, 36 were upregulated and 218 were downregulated (Figures 2B,C). Among the scanned miRNAs, 2 miRNAs (miR-551b and miR-448) were upregulated in both Treg-depleted groups, while 45 miRNAs were downregulated in both groups (Supplemental Figures 2A–C).

To verify the microarray results, we performed qRT-PCR assay to measure the changed miRNAs in four groups (CTL, CD25 mAb, Foxp3^DTR^-CTL, and Foxp3^DTR^-DT groups, 15 mice/group). The inclusion criteria of changed miRNAs was as follows: mean fold change >2 and *P*-value < 0.05 between Treg-depleted groups and control groups. Among the significantly changed miRNAs, we selected six to validate (Supplemental Figure 2, Figure 2D–I). Consequently, we identified that two miRNAs (miR-551b and miR-448) were significantly increased and four miRNAs (miR-9, miR-124, miR-148, and miR-34c) were markedly decreased in serum from Treg-depleted mice.

### Diagnostic value of the selected serum miRNAs

**Figure 3:**
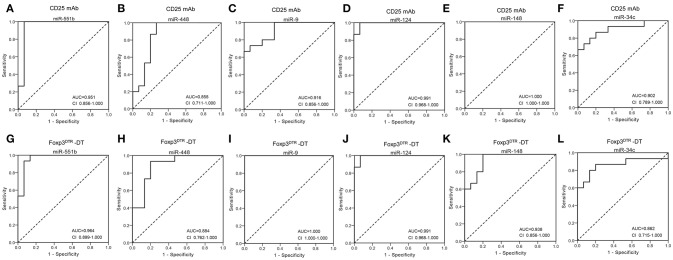
Diagnostic value of selected serum miRNAs. **(A–F)** ROC curve for the ability of individual miRNAs (miR-551b, miR-448, miR-9, miR-124, miR-148, and miR-34c) to separate CD25 mAb mice from controls. **(G–L)** ROC curve for the ability of miR-551b, miR-448, miR-9, miR-124, miR-148, and miR-34c to separate Foxp3^DTR^ –DT mice from controls.

Next, we conducted receiver-operating characteristic (ROC) curve analyses to identify the diagnostic usefulness of the 6 miRNAs for Treg-depletion mice models. ROC curve analysis revealed that the six miRNAs (miR-551b, miR-448, miR-9, miR-124, miR-148, and miR-34c) could serve as valuable biomarkers for distinguishing CD25 mAb samples from controls, with the AUC (the area under the ROC curve) values being 0.951, 0.858, 0.916, 0.991, 1.000, and 0.902, respectively (Figures 3A–F). Likewise, the ROC curves also indicated that the six miRNAs (miR-551b, miR-448, miR-9, miR-124, miR-148, and miR-34c) could accurately discern DT samples from controls, with the AUCs being 0.964, 0.884, 1.000, 0.991, 0.938, and 0.862, respectively (Figures 3G–L). The results suggest that the diagnostic potential of these six miRNAs in distinguishing Treg-depleted mice from controls was high.

### Microarray-based go and KEGG analyses revealed the role of the selected serum miRNAs

**Figure 4:**
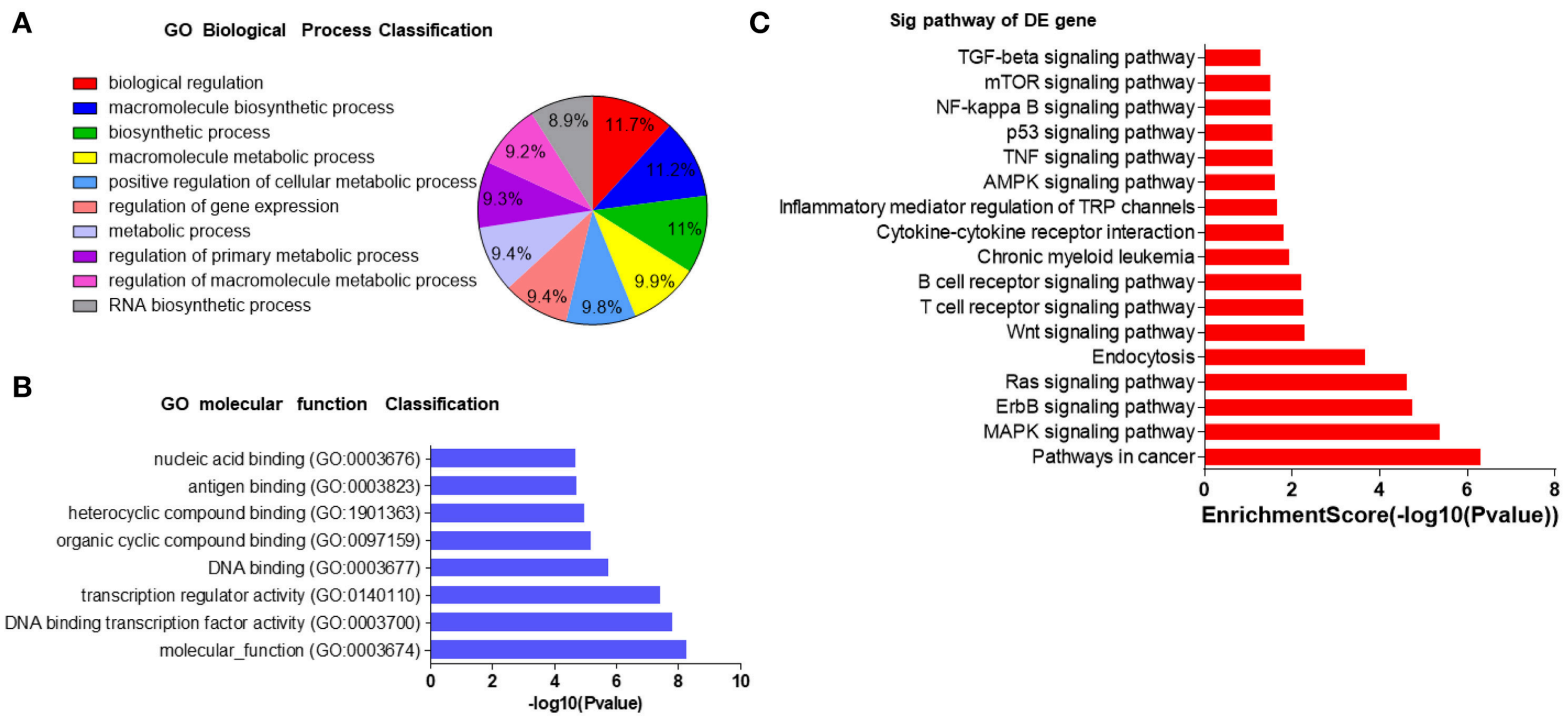
GO and KEGG analyses of potential roles of selected serum miRNAs. **(A,B)** The most enriched GO biological processes and molecular functions of 6 selected miRNAs. GO, molecular function for all miRNA targets. **(C)** Pathway enrichment analysis based on the miRNA target genes. The vertical axis represents the pathway category and the horizontal axis represents the enrichment score of the pathways and KEGG pathway terms (*P* < 0.05 and FDR < 0.05).

In order to understand the potential functions of these miRNAs, we conducted bioinformatics analysis. First, Gene Ontology (GO) analysis was performed to identify biological processes associated with the miRNA target genes (*P* < 0.001, FDR < 0.05). The high-enrichment GO terms targeted by the six miRNAs included biological regulation, macromolecule biosynthetic process, biosynthetic process and metabolic process (Figures 4A,B). KEGG annotation showed that oncogenic pathways (pathways in cancer, chronic myeloid leukemia, and the TNF signaling pathway), immune-associated pathways (T cell and B cell receptor signaling pathways, inflammatory mediator regulation of TRP channels, TGF-beta signaling pathway, cytokine-cytokine receptor interaction, and NF-kappa B signaling pathway), and important proliferative, survival, and apoptosis signaling pathways (MAPK, AMPK, ErbB, Ras, Wnt, mTOR, and p53) were significantly enriched (Figure 4C). Most of the pathways have already been reported to take part in immunodeficiency. For example, RAS-MAPK signaling pathway deregulation in T lymphocytes was found to result in a previously unknown primary immunodeficiency disease (33), mTOR pathway played a crucial part in regulating lymphoproliferation and aberrant differentiation in autoimmune lymphoproliferative syndrome (ALPS) (34), and the pivotal role of Wnt signaling pathway in T cell development, activation, and differentiation has recently been discovered (35). These bioinformatics interpretations may provide more evidence that the six miRNAs may have regulatory effects on immunity by affecting signaling pathways.

### Separation of patients with autoimmune diseases from controls by miR-448, miR-124, and miR-551b

**Figure 5:**
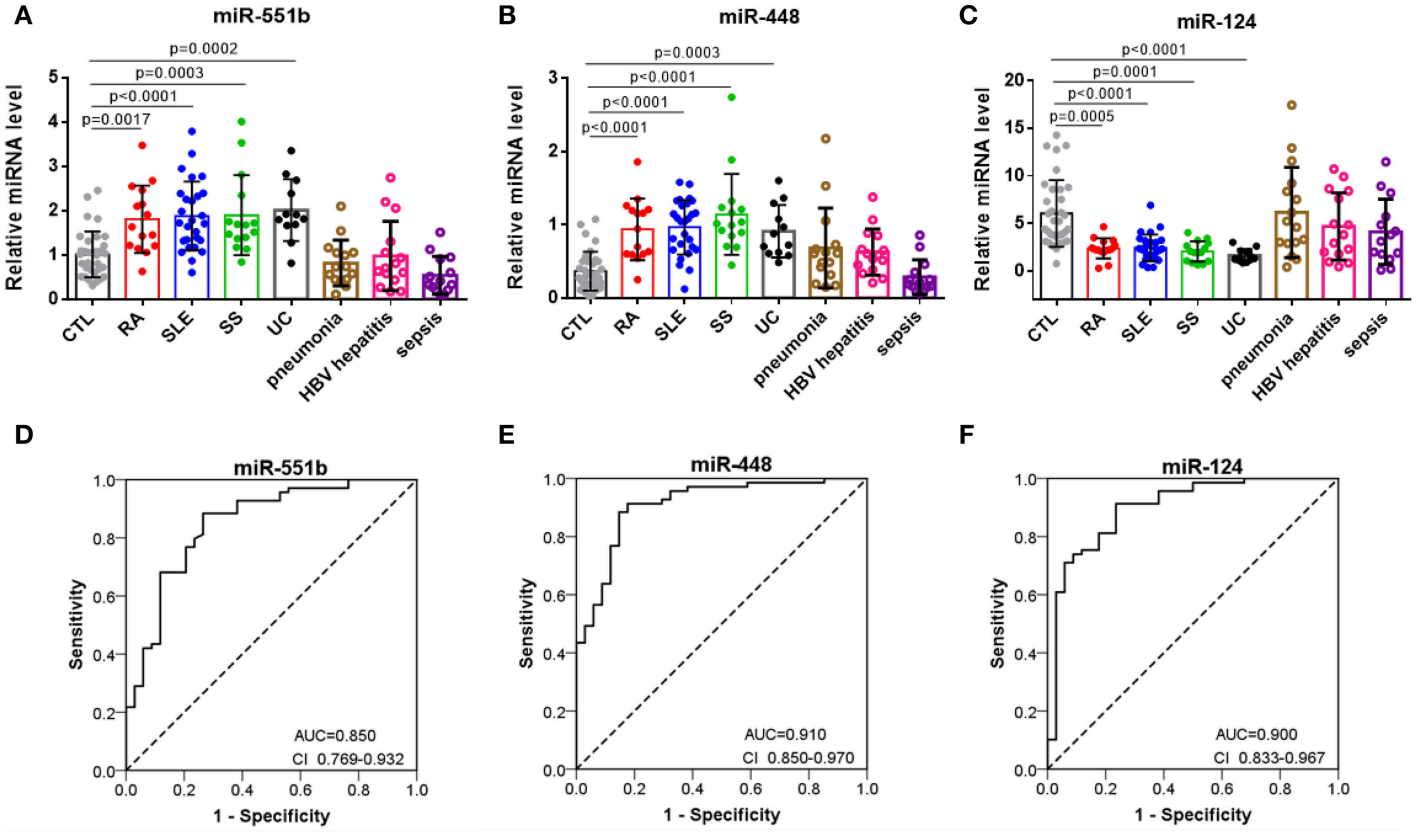
Separation of patients with autoimmune diseases from controls by miR-551b, miR-448, and miR-124. **(A–C)** The relative expressions of 3 miRNAs were studied in serum from 34 healthy controls, 15 RA patients, 27 SLE patients, 15 SS patients, 12 UC patients, 15 pneumonia patients, 15 HBV hepatitis patients, and 14 sepsis patients. **(D–F)** ROC curves for the ability of miR-551b, miR-448, and miR-124 to separate patients with autoimmune diseases from controls. All the values are shown as the mean ± SEM.

To further assess the diagnostic value of miRNA signatures in distinguishing patients with autoimmune diseases from controls, we measured the six miRNAs in serum samples comprising 34 healthy controls, 15 RA patients, 27 SLE patients, 15 SS patients, 12 UC patients. Supplemental Tables summarizes the demographic characteristics for the participants. QRT-PCR results indicated that miR-551b and miR-448 were significantly increased in RA, SLE, SS, and UC patients (Figures 5A,B), whereas miR-124 levels were decreased in RA, SLE, SS, and UC patients compared to the controls (Figure 5C). To further verify the three miRNAs are specific for systemic autoimmune diseases, we measured them in serum samples of inflammatory disease comprising 15 pneumonia patients, 15 HBV hepatitis patients, and 14 sepsis patients (Figures 5A–C). The results showed no obvious differences between them and healthy controls, suggesting that the three miRNAs may represent specific biomarkers for distinguishing patients with autoimmune diseases from healthy controls.

Then we performed a ROC curve analysis to evaluate the diagnostic usefulness of the three miRNA in discriminating patients with autoimmune diseases from healthy controls. The ROC curve analysis showed that miR-448, miR-124, and miR-551b could serve as valuable biomarkers for distinguishing patients with autoimmune diseases from healthy controls, with the AUC being 0.91(95% CI 0.85–0.97), 0.9 (95% CI 0.833–0.967), and 0.850 (95% CI 0.769–0.932), respectively (Figures 5D–F). Then, we analyzed the predictive accuracy of miRNA signatures: miR-448 showed a specificity of 82.4% and a sensitivity of 91.3%, miR-124 showed a specificity of 76.5% and a sensitivity of 91.3%, and miR-551b showed a specificity of 73.5% and a sensitivity of 88.4%. These results suggest that the diagnostic value of these three miRNAs to distinguish patients with autoimmune diseases from healthy individuals was high.

## Discussion

Autoimmune diseases involve a complicated immunity disorder, leading to a loss of self-tolerance and following assault on endogenous tissues and cells. So far, there is no universal biomarkers for detecting almost all autoimmune diseases.

Tregs play essential roles in maintaining immune homeostasis and preventing autoimmunity induced by excessive immune activation (10). Depletion of Tregs in mice is considered a representative animal model of autoimmune disease. In this study, we established Treg depletion mice models in two ways: through CD25 mAb injection in C57BL/6 mice and DT injection in Foxp3^DTR^ mice. Both models showed significantly reduced Treg levels and increased CD4^+^ T cell levels in peripheral blood and spleen. Inflammatory cytokines, such as TNF-α, IL-6, and IFN-γ, were markedly increased in serum from Treg-depleted mice. These phenomena are consistent with the common characteristics of autoimmune diseases, so these Treg-depleted mice can be used as representative models of autoimmune diseases.

In a previous study, we showed that miRNAs are present in serum and plasma of humans and many other animals with stable, reproducible, and consistent in the serum of individuals of the same species (16). By characterizing serum miRNA expression profiles under normal conditions and in various disease states, we found that serum miRNAs are derived not only from circulating blood cells but also from other tissues directly affected by diseases. Thus, we concluded that serum miRNAs can serve as potential biomarkers for detecting various diseases (16, 17, 36). Here, we first investigated the serum miRNA profiles in animal models with Treg depletion. Low Density Array identified miRNAs with significantly different levels in Treg-depleted mice and control mice and revealed that two miRNAs (miR-551b and miR-448) were upregulated and 45 miRNAs were downregulated (>2-fold change) in both Treg-depleted groups. QRT-PCR further confirmed that miR-551b and miR-448 were significantly increased and four miRNAs (miR-9, miR-124, miR-148, and miR-34c) were significantly decreased in Treg-depleted groups. Then, ROC curve analysis determined that 6 miRNAs (miR-551b, miR-448, miR-9, miR-124, miR-148, and miR-34c) could serve as valuable biomarkers for distinguishing Treg-depleted mice from controls. GO term and KEGG pathway annotation showed that target genes of the six miRNAs were associated with oncogenic, immune-associated, proliferative, survival, apoptosis, and inflammatory signaling pathways, and most of the pathways have already been reported to take part in immunodeficiency. Previous studies have reported that miR-551b is deregulated in CD (coeliac disease, a common autoimmune disorder of the small bowel) patients (37). Wu et al. (38) found that miR-448 is deregulated in MS patients and further promotes MS development through induction of the Th17 response. miR-9 has been found to be a putative GA-treatment responsive miRNA biomarker in EAE (experimental autoimmune encephalomyelitis) (39) and sympathetic ophthalmia (40). Previous research has demonstrated that miR-124 plays vital roles in regulating autoimmune inflammation (41–46). miR-148 might represent prognostic markers for treating autoimmune disorders, such as chronic inflammatory diseases, multiple types of cancer and heart failure in diabetics (47, 48). Besides, miR-34 has been reported to be correlated with RA (49).

Then, we measured six miRNAs to verify in the serum of RA, SLE, SS, UC patients, non-autoimmune diseases patients and healthy controls. QRT-PCR confirmation and ROC curve analysis determined that miR-448, miR-124, and miR-551b could serve as valuable specific biomarkers for distinguishing patients with autoimmune diseases from healthy controls.

In conclusion, we have defined a serum miRNA profiling in an animal model with autoimmune diseases. Moreover, our findings may provide a potential biomarker for diagnosing autoimmune diseases.

## Ethics statement

All animal care and handling procedures were performed in accordance with the National Institutes of Health's Guide for the Care and Use of Laboratory Animals and were approved by the Institutional Review Board of Nanjing University.

## Author contributions

XC, HZ, and YW conceived and designed the study. FJ, HH, MX, and SZ participated in the experiments and drafted the manuscript. HZ contributed to the sample collection and interpretation the data. All authors read and approved the final manuscript.

### Conflict of interest statement

The authors declare that the research was conducted in the absence of any commercial or financial relationships that could be construed as a potential conflict of interest.
