# Supplementary material for: Serum microRNA Profiles Serve as Novel Biomarkers for Autoimmune Diseases
Source: Front Immunol. 2018 Oct 16;9:2381. doi: 10.3389/fimmu.2018.02381 (PMC6232775; doi:10.3389/fimmu.2018.02381)
Supplement: Supplementary file 1 [file Data_Sheet_1.PDF]

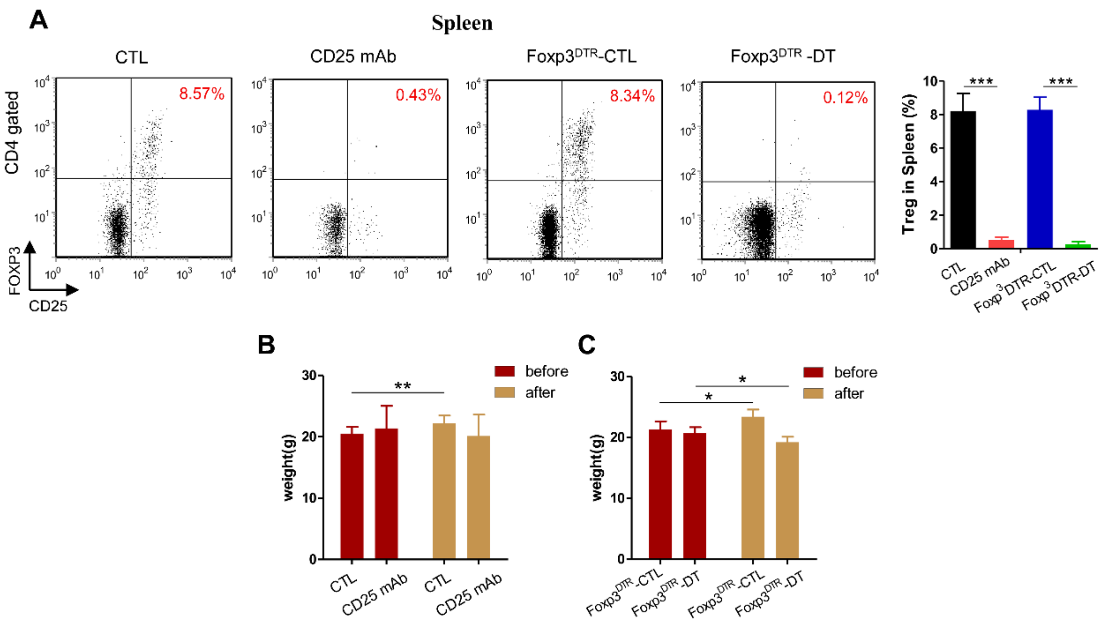

Supplemental Fig.1 Anti-CD25 mAb and Diphtheria toxin (DT) depletes CD4$^+$ CD25$^+$ Foxp3$^+$ Treg cells in spleen. The C57BL/6 and Foxp3^{DTR} mice were divided into two groups, 15/group. Then, C57BL/6 mice were administered with PBS or CD25 mAb every 3 days, Foxp3^{DTR} mice were continuously injected with DT for 7 days. At day 8, all mice were sacrificed, spleen were collected. **(A)** Analysis of CD4$^+$ CD25$^+$ Foxp3$^+$ Tregs in spleen. **(B, C)** The statistical analysis of the weights of mice from four groups. All the values are shown as mean ± SEM. *P < 0.05, **P < 0.01, and ***P < 0.005 (n = 15).

A

Down-regulated miRNAs				
mmu-miR-205	mmu-miR-130a	mmu-miR-148a	mmu-miR-433	mmu-miR-211
rno-miR-344-3p	mmu-miR-143	mmu-miR-135a	mmu-miR-199a-3p	mmu-miR-182
mmu-miR-194	mmu-miR-137	mmu-miR-193	mmu-miR-494	mmu-miR-199a-5p
mmu-miR-196b	rno-miR-381	mmu-miR-708	mmu-miR-872	mmu-miR-142-5p
mmu-miR-384-3p	mmu-miR-598	mmu-miR-546	mmu-miR-134	mmu-miR-203
mmu-miR-9	mmu-miR-376b	mmu-miR-124	mmu-miR-376c	mmu-miR-202-5p
mmu-miR-129-3p	mmu-miR-369-5p	mmu-miR-34c	mmu-miR-337-3p	mmu-miR-489
mmu-miR-489	mmu-miR-136	mmu-miR-302c	mmu-miR-544	mmu-miR-147
mmu-miR-208b	mmu-miR-107	mmu-miR-96	mmu-miR-207	mmu-miR-98

B

Up-regulated miRNAs
mmu-miR-551b
mmu-miR-448

C

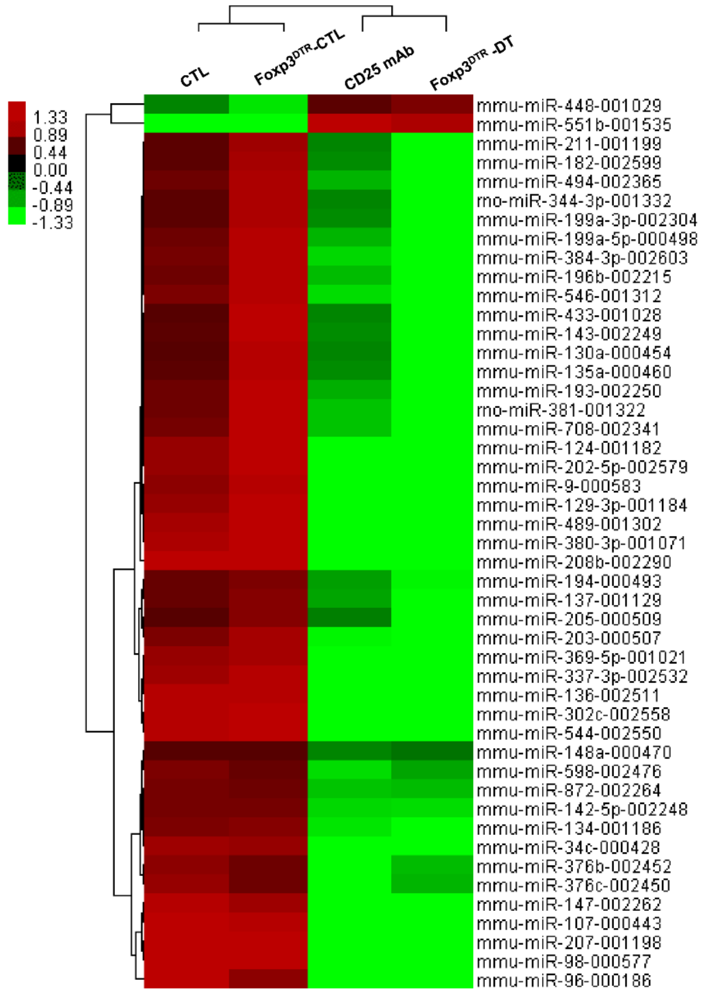

Supplemental Fig.2 Differentially expressed miRNAs in mouse models for Treg depletion. (A) Common downregulated miRNAs, **(B)** common upregulated miRNAs, **(C)** Hierarchical clustering analysis of common changed miRNAs in the serum of CD25 mAb and Foxp3^{DTR}–DT mice.

Supplemental Table 1. Clinical features of healthy subjects.

Number	Age	Gender		Number	Age	Gender	
1	62	F	healthy	18	63	M	healthy
2	15	F	healthy	19	21	M	healthy
3	20	M	healthy	20	62	M	healthy
4	48	F	healthy	21	72	F	healthy
5	51	F	healthy	22	38	F	healthy
6	51	F	healthy	23	33	M	healthy
7	25	F	healthy	24	49	F	healthy
8	67	M	healthy	25	56	F	healthy
9	42	F	healthy	26	32	F	healthy
10	35	F	healthy	27	39	F	healthy
11	40	M	healthy	28	45	M	healthy
12	67	F	healthy	29	66	M	healthy
13	36	M	healthy	30	58	F	healthy
14	60	F	healthy	31	60	F	healthy
15	66	M	healthy	32	47	M	healthy
16	47	F	healthy	33	42	M	healthy
17	32	F	healthy	34	21	M	healthy

Supplemental Table 2. Clinical and demographic characteristics of RA patients.

Number	Age	Gender	Pathological diagnosis	stage	DAS28
1	58	M	RA	active	2.9
2	53	F	RA	active	5.83
3	38	F	RA	active	3.15
4	60	F	RA	active	8.13
5	53	M	RA	active	6.07
6	65	F	RA	active	4.98
7	62	F	RA	active	5.73
8	72	F	RA	active	7.28
9	74	F	RA	active	5.03
10	52	F	RA	active	6.35
11	59	F	RA	active	3.97
12	68	F	RA	active	3.11
13	72	F	RA	active	7.15
14	72	F	RA	active	3.44
15	54	F	RA	active	8.27

Supplemental Table 3. Clinical and demographic characteristics of SLE patients.

Number	Age	Gender	Pathological diagnosis	stage	SLEDAI
1	30	F	SLE	active	7.08
2	24	M	SLE	active	19.49
3	43	F	SLE	active	6.96
4	35	F	SLE	active	15.52
5	35	M	SLE	active	10.91
6	27	F	SLE	active	9.54
7	37	F	SLE	active	14.84
8	16	F	SLE	active	13.67
9	29	F	SLE	active	9.89
10	44	F	SLE	active	13.83
11	28	F	SLE	active	14.72
12	61	F	SLE	active	16.27
13	37	F	SLE	active	7.83
14	36	M	SLE	active	7.27
15	45	F	SLE	active	23.3
16	56	F	SLE	active	13.84
17	42	F	SLE	active	26.93
18	46	F	SLE	active	15.05
19	47	F	SLE	active	24.89
20	41	F	SLE	active	23.76
21	39	F	SLE	active	8.55
22	37	F	SLE	active	10.93
23	13	F	SLE	active	7.86
24	20	F	SLE	active	9.56
25	42	F	SLE	active	18.57
26	24	F	SLE	active	15.83
27	45	F	SLE	active	18.56

Supplemental Table 4 Clinical and demographic characteristics of SS patients.

Number	Age	Gender	Pathological diagnosis
1	44	F	SS
2	78	F	SS
3	55	F	SS
4	54	F	SS
5	56	F	SS
6	50	F	SS
7	48	F	SS
8	52	F	SS
9	44	F	SS
10	65	F	SS
11	54	F	SS
12	61	F	SS
13	35	F	SS
14	52	F	SS
15	67	F	SS

Supplemental Table 5. Clinical and demographic characteristics of UC patients.

Number	Age	Gender	Pathological diagnosis
1	28	M	UC
2	29	F	UC
3	29	M	UC
4	26	F	UC
5	30	F	UC
6	24	M	UC
7	26	M	UC
8	75	M	UC
9	38	F	UC
10	25	F	UC
11	32	F	UC
12	48	F	UC
13	70	M	UC
14	76	F	UC
15	67	F	UC

Supplemental Table 6. Clinical and demographic characteristics of pneumonia patients.

Number	Age	Gender	Pathological diagnosis
1	36	F	Pneumonia
2	72	M	Pneumonia
3	50	F	Pneumonia
4	34	F	Pneumonia
5	47	F	Pneumonia
6	52	M	Pneumonia
7	56	M	Pneumonia
8	72	F	Pneumonia
9	82	M	Pneumonia
10	25	M	Pneumonia
11	34	M	Pneumonia
12	45	M	Pneumonia
13	60	F	Pneumonia
14	46	F	Pneumonia
15	67	F	Pneumonia

Supplemental Table 7.Clinical and demographic characteristics of HBV hepatitis patients.

Number	Age	Gender	Pathological diagnosis
1	48	M	Chronic Hepatitis B hepatitis
2	52	M	Chronic Hepatitis B hepatitis
3	67	M	Chronic Hepatitis B hepatitis
4	52	F	Chronic Hepatitis B hepatitis
5	46	F	Chronic Hepatitis B hepatitis
6	64	M	Chronic Hepatitis B hepatitis
7	59	M	Chronic Hepatitis B hepatitis
8	60	F	Chronic Hepatitis B hepatitis
9	64	M	Chronic Hepatitis B hepatitis
10	39	M	Chronic Hepatitis B hepatitis
11	38	F	Chronic Hepatitis B hepatitis
12	46	M	Chronic Hepatitis B hepatitis
13	58	M	Chronic Hepatitis B hepatitis
14	61	M	Chronic Hepatitis B hepatitis
15	46	F	Chronic Hepatitis B hepatitis

Supplemental Table 8. Clinical and demographic characteristics of sepsis patients.

Number	Age	Gender	Pathological diagnosis
1	32	M	sepsis
2	54	M	sepsis
3	68	F	sepsis
4	51	F	sepsis
5	49	M	sepsis
6	50	M	sepsis
7	48	F	sepsis
8	52	F	sepsis
9	47	F	sepsis
10	59	M	sepsis
11	62	M	sepsis
12	64	M	sepsis
13	45	M	sepsis
14	38	F	sepsis
